# Developmental cues and persistent neurogenic potential within an *in vitro *neural niche

**DOI:** 10.1186/1471-213X-10-5

**Published:** 2010-01-14

**Authors:** Chris Pierret, Jason A Morrison, Prakash Rath, Rachel E Zigler, Laura A Engel, Corinne L Fairchild, Huidong Shi, Joel A Maruniak, Mark D Kirk

**Affiliations:** 1 Division of Biological Sciences, University of Missouri, Columbia, MO 65211, USA; 2 Department of Pathology and Anatomical Sciences, University of Missouri School of Medicine, Columbia, MO 65212, USA; 3 Current address: Department of Biochemistry and Molecular Biology, Guggenheim 13, Mayo Clinic, 221 4th Ave. SW, Rochester, MN 55902, USA

## Abstract

**Background:**

Neurogenesis, the production of neural cell-types from neural stem cells (NSCs), occurs during development as well as within select regions of the adult brain. NSCs in the adult subependymal zone (SEZ) exist in a well-categorized niche microenvironment established by surrounding cells and their molecular products. The components of this niche maintain the NSCs and their definitive properties, including the ability to self-renew and multipotency (neuronal and glial differentiation).

**Results:**

We describe a model *in vitro *NSC niche, derived from embryonic stem cells, that produces many of the cells and products of the developing subventricular zone (SVZ) and adult SEZ NSC niche. We demonstrate a possible role for apoptosis and for components of the extracellular matrix in the maintenance of the NSC population within our niche cultures. We characterize expression of genes relevant to NSC self-renewal and the process of neurogenesis and compare these findings to gene expression produced by an established neural-induction protocol employing retinoic acid.

**Conclusions:**

The *in vitro *NSC niche shows an identity that is distinct from the neurally induced embryonic cells that were used to derive it. Molecular and cellular components found in our *in vitro *NSC niche include NSCs, neural progeny, and ECM components and their receptors. Establishment of the *in vitro *NSC niche occurs in conjunction with apoptosis. Applications of this culture system range from studies of signaling events fundamental to niche formation and maintenance as well as development of unique NSC transplant platforms to treat disease or injury.

## Background

The mammalian brain contains a NSC niche that produces neurons and glia for the developing (within the SVZ) and adult (within the SEZ) brain [1-9]. This niche consists of NSCs, their progeny (transit amplifying cells and neuroblasts), ependymal cells, a basal lamina and complex extracellular matrix (ECM), and a microenvironment consisting of factors provided by cells within the niche and adjacent blood vessels [1,3,4,6,8-14]. The function of the niche is to produce new neurons and glia during development and to supply replacement cells due to turnover in the olfactory bulb and in response to disease or injury in the central nervous system [1-9]. Recent classification of an intermediate progenitor adjacent to the ventricular zone has made the nomenclature of neural stem and progenitor cells and respective niches more complex [15,16]. Reference to related literature is here made by maintaining the terminology used therein whenever possible. For best anatomical reference, the subventricular zone (SVZ) refers to the area of the developing NSC niche and the subependymal zone (SEZ) refers to the adult NSC niche. The authors will make every effort to further relate the timeframe and location.

Components of the ECM in the embryonic ventricular zone play a vital role in maintenance of a NSC population [17,18], and may direct the fate of stem cells and their progeny in the developing brain [17-20]. Laminin, nidogen 1 (entactin), perlecan, fibronectin, collagen IV, syndecan and other components of the ECM are necessary for central nervous system (CNS) development [3,17-19]. Receptors for ECM subunits, including integrins (specifically the β-1 subunit) appear to be specifically regulated in stem cells within the niche and likely contribute to stem cell self-renewal by physically tethering these cells in preparation for asymmetric division [3]. Cadherins are temporally regulated in embryonic neural development to specific patterns in the ventricular zone [17], potentially indicating a role in stem cell maintenance.

Transcription factors (TFs) regulate and define the process of neurogenesis in embryos and adults [21,22]. For instance, a progressive sequence of TF expression, starting with Pax6 and including Eomes, NeruoD, and Tbr1, highlight the developmental timeline for production of projection neurons in the developing neocortex [22,23].

During development and in adulthood, apoptosis plays a vital role in neurogenesis [24]. Cells within germinal centers, such as the SVZ, show particularly high levels of apoptosis as excess cells are eliminated to allow targeting and further differentiation of appropriately networked neurons and glia [24,25]. The vertebrate brain loses 70% of its neurons due to apoptosis during development [24,26].

The *in vitro *derivation of neural cells from mammalian embryonic stem cells has been achieved using various induction protocols [27-33]. The neurogenic potential and cellular phenotypes derived from these methods are varied [30]. Retinoic acid induction of embryonic stem cells results in a high proportion of neural cells [34]. However, it yields many post-mitotic neurons and glia, preserving relatively few neural stem or progenitor cells [35].

Recently, we described a culture system in which retinoic acid neural induction is followed by plating at high-density, resulting cell cultures that display elements of an *in vitro *NSC niche [9]. Using this protocol, we identified putative NSCs within the aggregates contained in the cultures. These NSCs co-expressed platelet-derived growth factor receptor-alpha (PDGFRα) and glial fibrillary acidic protein (GFAP), indicative of the NSC (B cell) within the SEZ NSC niche [6]. NSCs from the *in vitro *NSC niche could be propagated as neurospheres with retention of their neural potential after four passages [9].

Here, we demonstrate apoptotic activity concurrent with the development of the *in vitro *NSC niche, application of our culture system to a 3-D scaffold, and provide a quantitative comparison of the cells and cell products of the *in vitro *NSC niche to those resulting from the original 4-/4+ retinoic acid protocol [34].

## Results

### Electron microscopy reveals presumptive neuroblasts within multicellular aggregates

**Figure 1:**
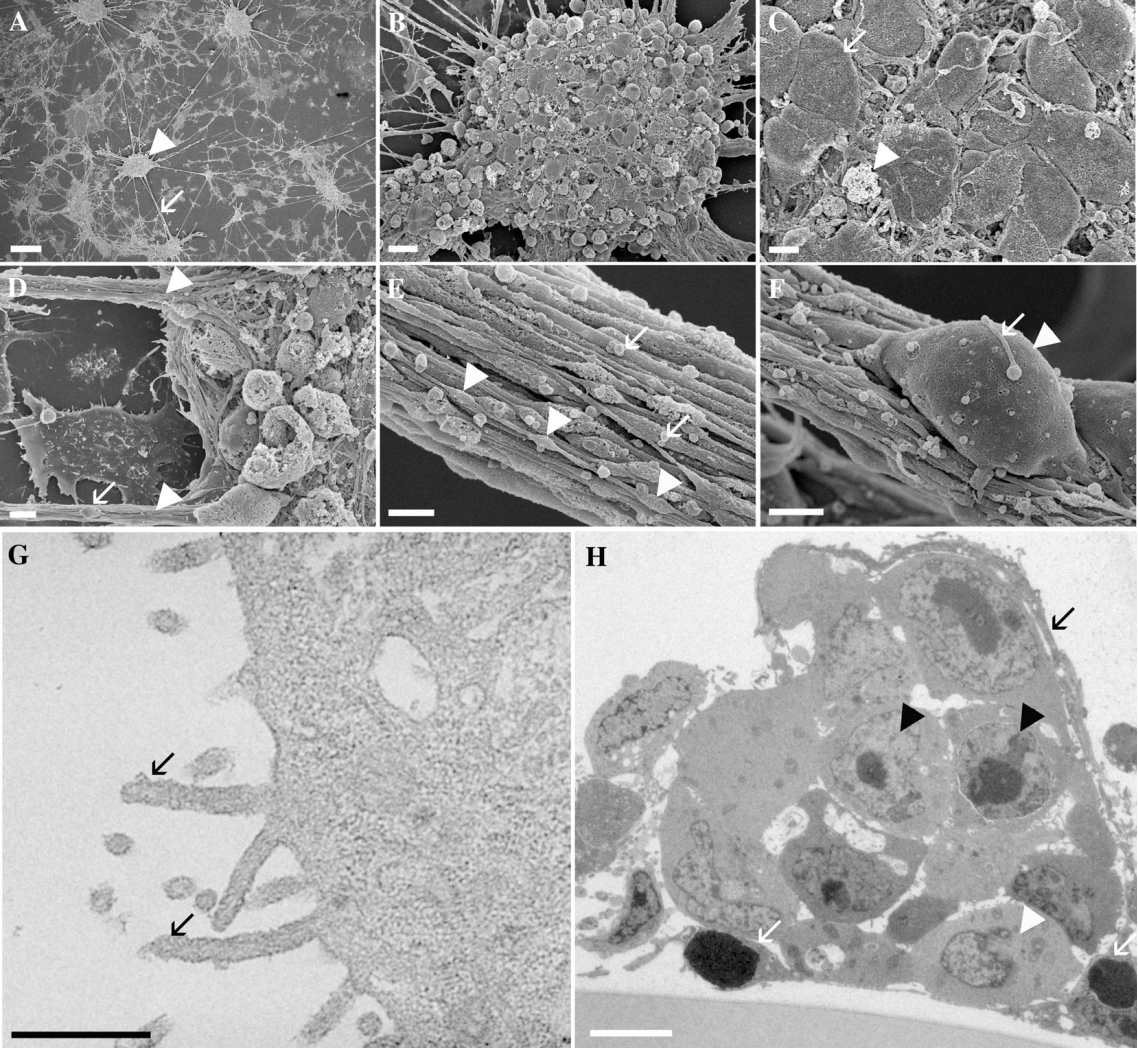
**Scanning and Transmission Electron Microscopy (SEM and TEM) of the Day 14 *in vitro *NSC niche**. **A**, SEM micrograph shows multicellular aggregates (arrowhead) and processes (arrow) between aggregates. Scale bar is 100 μm. **B**, Higher magnification of an aggregate shows many processes attached to a single aggregate. Scale bar is 10 microns. **C**, Cells of the aggregate shown in **B **include those with intact (arrow) and apoptotic (arrowhead) appearance. Scale bar is 3 μm. **D**, Cells (arrow) can be seen attached to processes (arrowheads). Scale bar is 3 μm. **E**, Magnification of **D **reveals the presence of varicosities (arrowheads) and blebs (arrows). Scale bar is 1 micron. **F**, Magnification of **D **including a cell (arrowhead) attached to the process. A very small process (arrow) is present on top of the migratory cell. Scale bar is 2 μm. **G**, TEM micrograph of a cell from the edge of an aggregate shows microvilli (arrows) and intracellular organelles. Scale bar is 0.5 μm. **H**, TEM micrograph of a section from an aggregate suggests the presence of cell-types. Scale bar is 4 μm.

Mouse embryonic stem (ES) cells were induced with retinoic acid, dissociated, and plated on an entactin-collagen-laminin (ECL) coated surface at high density (250,000 cells/cm^2^) as we previously described [9]. After 6 days (Day 14 from start of induction) as adherent cultures in the absence of serum, the cells developed a reproducible structure with characteristics similar to those of a NSC niche [9]. Detailed morphology and ultrastructure of the Day 14 *in vitro *niche was obtained with scanning and transmission electron micrographs (Fig. 1). Scanning electron microscrographs elucidate the complex morphology within the compact multi-cellular aggregates that are positioned in a repeating pattern across the cultures and interconnected by processes [9]. Differentiated cells with flattened morphologies were observed in spaces between aggregates and below the inter-aggregate processes (Fig. 1A). Fig 1B and 1D-F shows details of the numerous multicellular strands extending from a complex aggregate. Some cells on the surface of this aggregate did not survive, likely due to prolonged exposure to the serum-free conditions (Fig. 1C).

The strands extending between aggregates were composed of many filamentous processes, and varicosities were evident along most of these processes (Fig. 1E). Cells that were apparently migrating between aggregates [9,31] exhibited microvilli (Fig. 1F) shown in higher detail by transmission electron micrographs (Fig. 1G). The interconnectivity of the interaggregate strands or processes (Fig. 1E-G) suggests a high level of long-distance interactions, while the presence of microvilli extending between adjacent cells indicates local intercellular interactions. In Fig. 1H, transmission electron microscopy (TEM) demonstrates the heterogeneity within the cellular morphologies of the cluster. An ultrathin section through a single aggregate revealed cellular morphologies typically found in the SEZ NSC niche. The long process in Fig. 1H (black arrow), dense nuclear staining, and high nuclear to cytoplasmic ratio (Fig. 1H, white arrow), are consistent with the properties of a neuroblast (equivalent to an A cell) [36,37]. Furthermore, the adjacent electron-lucent cell with nuclear invaginations (Fig. 1H, white arrowhead) may represent the B-like cell, while the more spherical cells (Fig. 1H, black arrowheads) likely represent C-like cells. Single sections of nineteen additional aggregates from two individually prepared cultures were examined and thirteen showed similarly identifiable A-, B- and C-like cells, three did not show the indicative cell morphologies, and three sections did not contain visible nuclei.

### Expression timeline of genes indicative of the cells of a NSC niche

**Figure 2:**
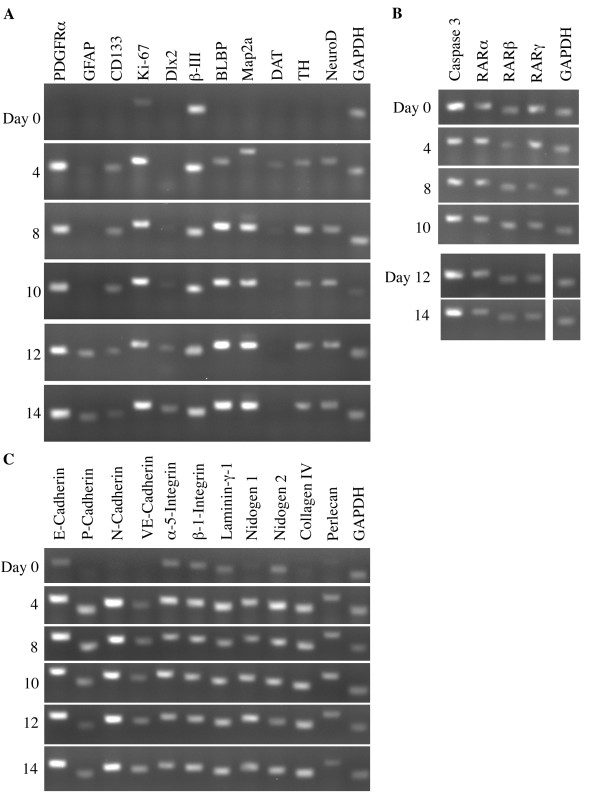
**RT-PCR profile of *in vitro *NSC niche by day of induction**. Day 0 represents undifferentiated mouse embryonic stem cells. Day 4 and Day 8 represent suspended embryoid bodies during 4-/4+ retinoic acid neural induction; Day 4 cells are harvested prior to the addition of retinoic acid, and Day 8 is harvested after 4 days of retinoic acid exposure. Days 10, 12 and 14 represent time-points of further neural induction as attached cultures on ECL. **A**, Neural markers during culture development. **B**, Retinoic acid receptors and caspase-3 through Day 14. (Days 12 and 14 and their housekeeping gene run together but on a second gel) **C**, Extracellular matrix (ECM) components and receptors during culture development.

Expression of genes related to neural development as well as neuronal and glial maturation was analyzed at sequential time-points during the development of our *in vitro *NSC niche (Fig. 2A). Undifferentiated ES cells (Day 0), suspended embryoid bodies (EBs) before and after retinoic acid neuralization (Day 4 and Day 8) and attached cells of the maturing culture system (Days 10, 12 and 14) were used to prepare whole cell lysates for mRNA isolation and RT-PCR analysis. Primer and reference sequences for each gene along with amplicon length are provided in Additional file 1. Expression of platelet-derived growth factor receptor alpha (PDGFRα) and glial fibrillary acidic protein (GFAP) suggest the presence of either mature oligodendrocytes and astrocytes, or NSCs [1,4-6,9]. Both of these markers were expressed in our mature NSC niche cultures (PDGFRα as early as Day 4, GFAP after Day 12) (Fig. 2A). CD133 (also known as mouse prominin-1) is expressed by cells in our cultures as early as Day 4 (Fig. 2A); CD133 expression may indicate neural stem or precursor cells or ependymal cells in embryonic murine neural tissue [38]. Proliferation, as indicated by Ki-67 expression, was present at all sampling points during *in vitro *NSC niche development (Fig. 2A).

Distal-less homeobox gene 2 (Dlx2) is a marker of the transition from NSC to transit-amplifying cell in the adult SEZ [4]. Dlx2 expression was present on and after Day 8 in our cultures (Fig. 2A). Neuronal differentiation in the *in vitro *NSC niche was indicated by expression of early neuronal marker β-III Tubulin, neuronal marker microtubule associated protein 2a (Map2a), neural development marker NeuroD, and the dopaminergic/catacholaminergic neuron marker tyrosine hydroxylase (TH) (Fig. 2A). Dopamine active transporter (DAT) expression, however, was not detectable in our cultures except on Days 4 and 8. Brain lipid binding protein (BLBP), a marker of radial glia, was present on and after Day 4 of culture.

Assessment of extracellular matrix (ECM) components and receptors that have been used as indicators of NSC niche development [17] revealed the following: A variety of cadherins and integrins were expressed at all sampling points after Day 0 (Fig. 2C), as well as their target ECM components, including the γ subunit of laminin, nidogen, perlecan, syndecan-3 and collagen IV. A member of the apoptotic cascade, caspase-3 [39], was expressed at all time-points tested, as were retinoic acid receptors α, β, and γ Fig. 2B). These receptors regulate subsets of target genes during retinoic acid-induced differentiation [40] and are involved in many aspects of neural and non-neural development [41].

### Cell plating density on Day 8 determines niche formation

Initial cell density used for plating on ECL-coated slides on Day 8 was consistently maintained at 250,000 cells/cm^2 ^for all experiments. Attempts to plate at higher densities (5 × 10^5^- 2 × 10^6 ^cells/cm^2^) resulted in failure of niche formation (Additional file 2). When plated at the latter, extremely high plating densities on Day 8 cells produced aggregates and interconnectivity between aggregates as early as Day 10, but the aggregates did not maintain attachment to the surface, apparently due to insufficient anchoring of the culture system to the exogenously applied ECL. This loss of attachment resulted in the break-up of aggregates and processes (data not shown). Plating cells on Day 8 at densities below 250,000 cells/cm^2 ^allowed attachment beyond Day 14, but compact aggregates and interconnective processes did not form (Additional file 2E).

### *In vitro *NSC niche in 3-D culture provides similar results

**Figure 3:**
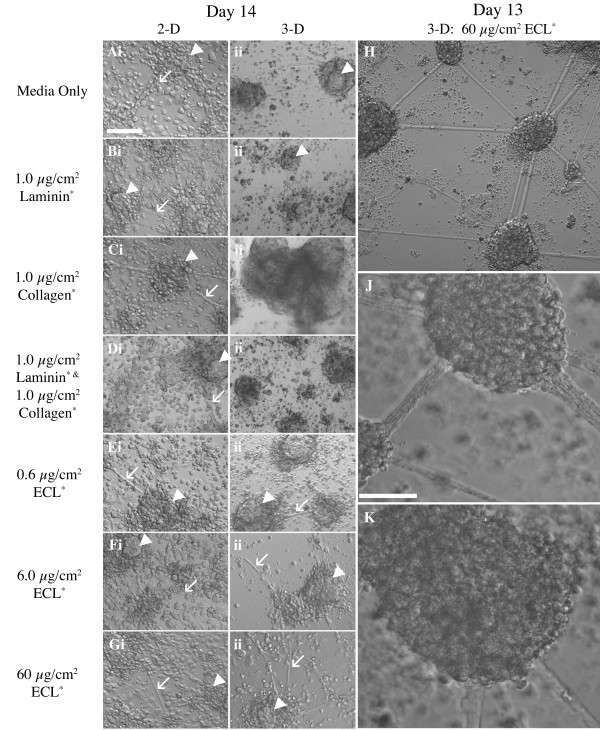
**Light microscopy of 2- and 3-D cultures grown in the presence of various ECM components**. **Ai-Gi**, Following 4-/4+ retinoic acid neural induction of mouse embryonic stem cells, the cells are plated in 96-well plates at 250,000 cells/cm^2 ^on the substrate as indicated. Images are from Day 14 cultures. **Aii-Gii**, Cells are plated in 3-D using 0.15% Puramatrix hydrogel with the addition of the ECM substrate as indicated. In all panels, arrows indicate process development and arrowheads indicate compact aggregate formation. Images are from Day 14 cultures. **H-K**, Higher magnification of Day 13 cultures grown in PuraMatrix hydrogel at 60 μg/cm^2 ^ECL reveals the best formation of *in vitro *NSC niche in 3-D of any conditions tested. All conditions tested produced *in vitro *neural niche in 2-D, but only 60 μg/cm^2 ^ECL is able to consistently support its formation in 3-D. Scale bar in **Ai **is 100 μm and applies to panels **Ai-H**. Scale bar in **J **is 50 μm and applies to **J **and **K**. * Concentrations of ECM components are expressed per surface area of the culture well. ECM components for 3-D cultures are added at the same concentration as 2-D cultures. The volume of PuraMatrix assembled from 50 μl of 0.15% PuraMatrix in each well increases the surface area for ECM attachment, thus diluting the effective concentration of the ECM components. Note the 10-fold increase in effective concentration of ECM components in 3-D cultures as compared to 2-D cultures.

To determine the effects of a more complex microenvironment on niche formation, we applied the *in vitro *NSC niche to a 3-D scaffold. PuraMatrix was diluted to 0.15% to promote neurite outgrowth as suggested by the manufacturer. ECM components laminin, collagen IV, or ECL were mixed with the PuraMatrix prior to scaffold self-assembly to provide anchoring sites for NSCs, similar to that established in 2-D culture [9]. On Days 10 and 12, morphological differences in the 2-D versus 3-D culture became apparent including lack of either aggregate or process formation in all but one of the seven 3-D ECM treatment groups (Additional file 3). This result was reproduced over 10 trials. Only a high ECL concentration (60 μg/cm^2^) resulted in 3-D cultures that were similar morphologically to our mature 2-D cultures (Fig. 3G and Additional file 3Oii). These data suggest a crucial role for components of the ECL in 3-D niche formation.

**Figure 4:**
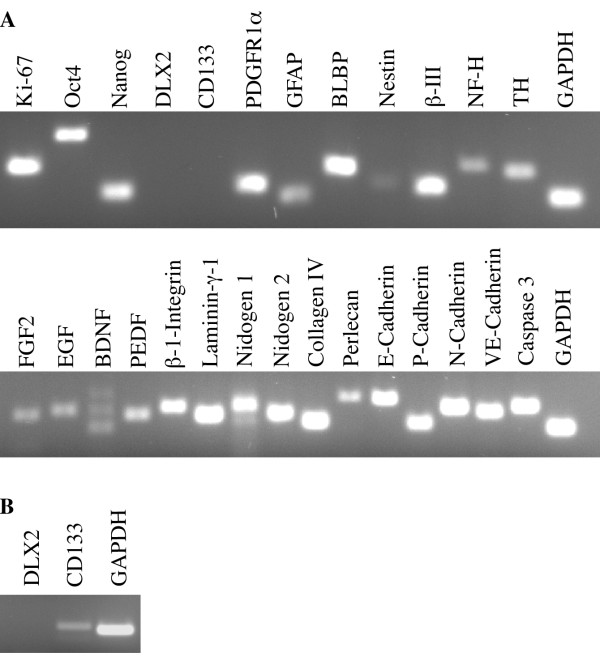
**RT-PCR of Day 14 3-D cultures**. **A**, Markers indicative of *in vitro *NSC niche development reveal that Dlx2 is not expressed at detectable levels in 3-D cultures. **B**, Increasing the total cDNA input for PCR reactions reveals low-level CD133 (but not Dlx2) expression in 3-D cultures.

Light micrographs at Days 13 and 14 (Fig. 3) revealed that the highest tested concentration of ECL (60 μg/cm^2^) resulted in 3-D cultures with structures equivalent morphologically to the 2-D *in vitro *NSC niche cultures. The 2-D cultures did not demonstrate dependence on the exogenous ECM components (Fig. 3A-G, left panels) for aggregate and process formation, though the cultures had the most consistent spacing of aggregates with 6.0 μg/cm^2 ^ECL applied to a 2-D surface (Fig. 3Fi, higher magnification in 3H, J, and 3K). RT-PCR was performed using Day 14, 3-D culture lysates to compare gene expression to Day 14, 2-D results shown in Fig. 2 and as we reported previously [9] (Fig. 4). Note that Dlx2 and CD133 were not detected in initial testing; however, CD133 expression was observed when cDNA input was doubled in the PCR reaction (Fig. 4B), suggesting that expression levels in some cases could be below that detectable by our assay.

RT-PCR for brain-derived neurotrophic factor (BDNF) detected splice variants (Fig. 4A), of which many have been previously documented [42]. 3-D cultures presented additional challenges, including small culture volume (PuraMatrix recommends 48-well culture plates or smaller) and difficulty in optimizing antibody-labeling techniques for the semi-fluid 0.15% scaffold. Growth of our cultures within the 3-D Puramatrix scaffold appeared similar to 2-D culture in aggregate formation (though at higher ECL concentration), process connectivity, and in the expression of many genes. However, because of the aforementioned challenges as well as the differences in Dlx2 and CD133 expression, we restricted the remaining work to 2-D culture.

### *In vitro *NSC niche in 2- and 3-D cultures integrate into organotypic brain slices

To demonstrate the ability of the 2- and 3-D cultures to integrate into brain tissue, cultures were harvested and applied to organotypic brain slice cultures. Eight days after transplantation, remaining cells were imaged. Both the 2- and 3-D cultures were capable of integration and survival in the brain tissue (Additional file 4).

### Apoptosis during niche formation

**Figure 5:**
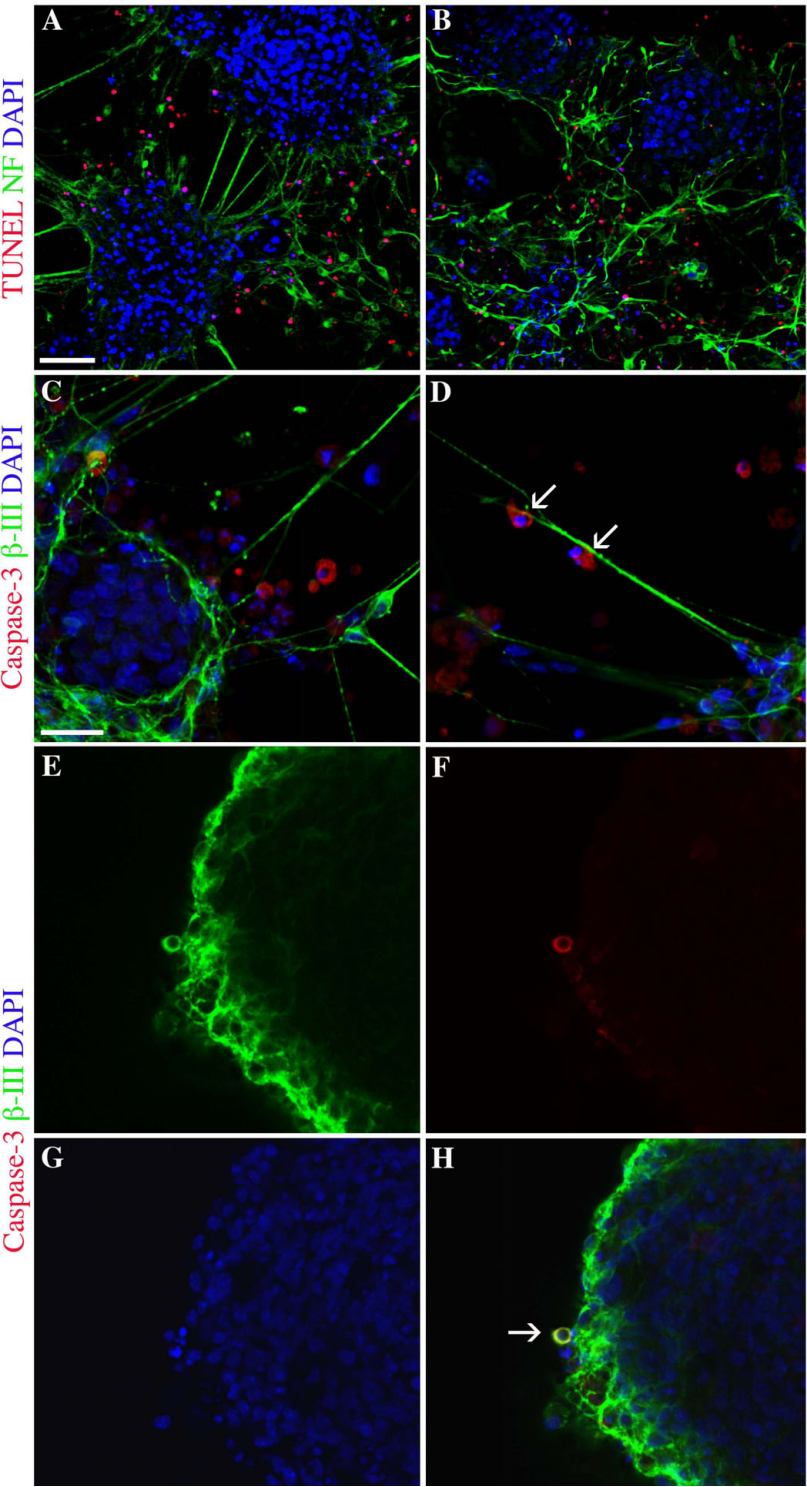
**Fluorescent antibody labelling indicates apoptotic cells in Day 14 *in vitro *NSC niche culture**. **A **and **B**, TUNEL (shown in red) indicates apoptotic cells within the culture are found at the outer edge of aggregates and in the area between aggregates. Neurafilament (NF) (shown in green) labeling suggests that the apoptotic cells are localized among mature cells of the culture. **C**, Activated caspase-3 (shown in red) labeling further indicates apoptotic cells in areas between aggregates. β-III tubulin^+ ^early neurons (in green) are indicated at the edge of the aggregate, within inter-aggregate processes and in the space between aggregates. **D**, Two cells expressing activated caspase-3 are shown along a neuronal process (labeled in green) between aggregates. **E-H**, Successive micrographs indicate a cell (arrow in **H**) that is positive for caspase-3 and β-III tubulin, indicating an apoptotic neuron. DAPI staining, shown in blue in all panels indicates nuclei. The scale bar in **A **is 50 μm and applies to **A **and **B**. Scale bar in **C **is 20 μm and applies to **C-H**.

TUNEL analysis and caspase-3 fluorescent-antibody labeling demonstrated that apoptotic cells were present in Day 14 cultures but were limited to the surfaces of aggregates and the spaces between aggregates (Fig. 5A-D). There was no indication that apoptosis occurred within the cores of the aggregates, and no examples of apoptotic NSCs were observed using double-label techniques with caspase-3 and PDGFRα or caspase-3 with GFAP. Examples of apoptotic neurons were identified (Fig. 5E-H).

### Differential gene expression in the *in vitro *NSC niche

**Figure 6:**
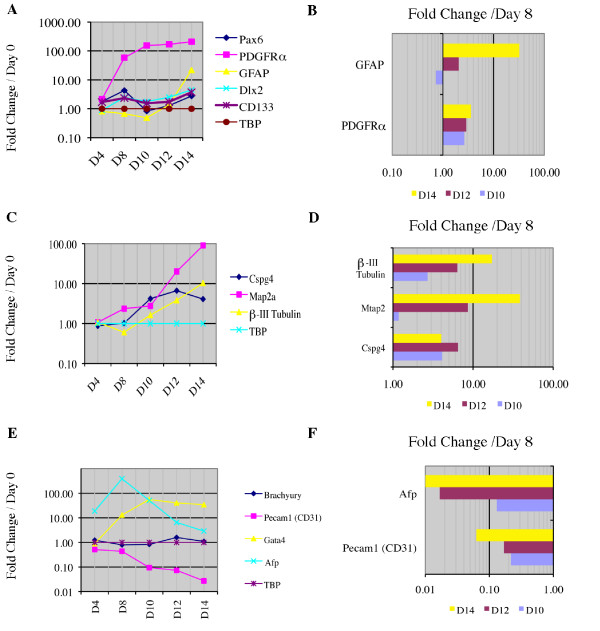
**Quantitative RT-PCR of genes indicative of particular developmental lineages**. **A, C **and **E**, Fold changes in expression levels of (**A**) NSC-associated genes, (**C**) mature neural cell-associated genes, (**E**) mesodermal and endodermal-associated genes in the developing *in vitro *NSC niche compared to Day 0 mouse embryonic stem cell cultures. **B, D **and **F**, Fold changes of (**B**) NSC-associated genes, (**D**) mature neural cell-associated genes, (**F**) mesodermal and endodermal-associated genes in the developing *in vitro *NSC niche compared to cells that have undergone 4-/4+ retinoic acid induction (Day 8). Of the genes tested (see methods) only those genes with statistically significant (p < 0.05) differences from Day 8 cultures are shown. TATA box binding protein (TBP) is used as the housekeeping gene in all experiments. Data supporting our housekeeping gene selection are provided in Additional file 6. Genes: Paired box gene 6 (Pax6), Platelet derived growth factor receptor alpha (PDGFRα), Glial fibrillary acidic protein (GFAP), Distal-less homeobox 2 (Dlx2), Prominin 1 (Prom1, also CD133), Chondroitin sulfate proteoglycan 4 (Cspg4), Microtubule-associated protein 2 (Map2a), β-III tubulin, Brachyury, Platelet/endothelial cell adhesion molecule 1 (Pecam1), GATA binding protein 4 (Gata4), Alpha fetoprotein (Afp) and TATA box binding protein (TBP).

**Figure 7:**
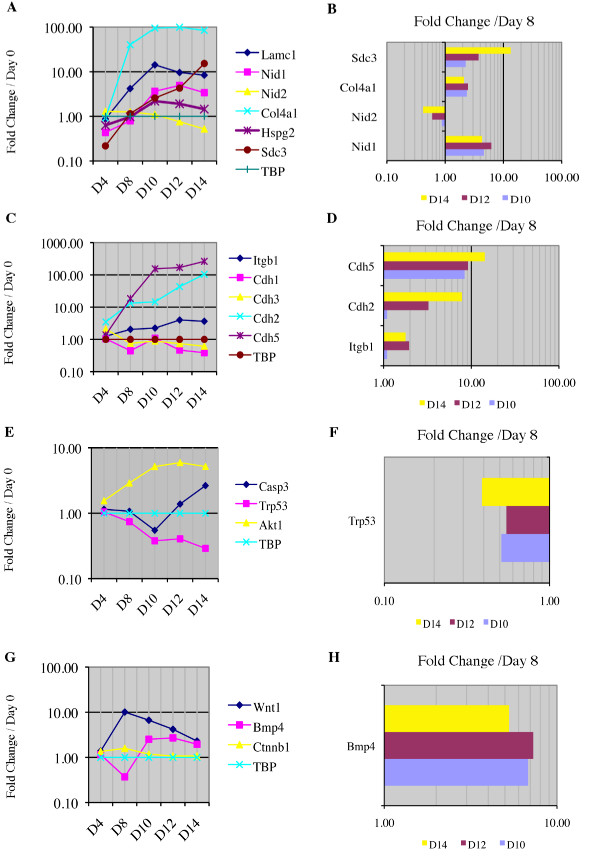
**Quantitative RT-PCR of genes indicative of extracellular matrix proteins and receptors, apoptosis and Wnt signalling**. **A, C, E **and **G**, Fold change of (**A**) extracellular matrix (ECM)-associated genes, (**C**) ECM receptor genes, (**E**) apoptosis-associated genes and (**G**) Wnt canonical pathway-associated genes in the developing *in vitro *NSC niche are compared to Day 0 mouse embryonic stem cell cultures. **B, D, F **and **H**, Fold change of (**B**) extracellular matrix-associated genes, (**D**) ECM receptor genes, (**F**) apoptosis-associated genes and (**H**) Wnt canonical pathway-associated genes in the developing *in vitro *NSC niche are compared to cells that have undergone 4-/4+ retinoic acid induction (Day 8). Only genes with statistically significant (p < 0.05) differences from Day 8 cultures are shown. TATA box binding protein (TBP) is used as the housekeeping gene in all experiments. Data supporting our housekeeping gene selection are provided in Additional file 6. Genes: Laminin, gamma 1 (Lamc1), Nidogen 1 (Nid1), Nidogen 2 (Nid2), Procollagen, type IV, alpha1 (Col4a1), Perlecan (Hspg2), Syndecan-3 (Sdc3), beta-1 Integrin (Itgb1), Cadherin 1 (Cdh1, E-cadherin), Cadherin 3 (Cdh3, P-cadherin), Cadherin 2 (Cdh2, N-cadherin), Cadherin 5 (Cdh5, VE-cadherin), Caspase-3 (Casp3), Transformation related protein 53 (Trp53), thymoma viral proto-oncogene 1 (Akt1), Wingless-related MMTV integration site 1 (Wnt1), Bone morphogenetic protein 4 (Bmp4), beta-1 Catenin, (Ctnnb1) and TATA box binding protein (TBP).

Custom SuperArray RNA profiler plates were designed to study mRNA expression throughout neural induction and niche formation to compare our *in vitro *NSC niche to retinoic acid induction alone. Categories used to compare expression between the niche and retinoic acid induction alone included genes indicative of: (1) neural stem and progenitor cells, (2) mature neural phenotypes, (3) mesodermal and endodermal lineages, (4) ECM components and receptors, (5) apoptosis, and (6) the canonical Wnt pathway. The genes, their abbreviations and transcript sequence used in the custom SuperArray plates are described in Additional file 5. The left panels in Fig. 6 and 7 show the progression of all genes relative to Day 0 and normalized to the expression of the TATA box binding protein (TBP), a housekeeping gene. The right panels of Fig 6 and 7 show those genes that were differentially expressed relative to Day 8 (discussed below). Twelve housekeeping genes were compared for consistency of gene expression across a subset of samples prior to selection of TBP for the primary experiments. The comparison of housekeeping genes is detailed in Additional file 6.

Gene expression indicative of neurogenesis is shown relative to undifferentiated ES cells in Fig 6A and 6C. This expression pattern is compared to Day 8 (RA induction alone) in Fig. 6B and 6D. Next, indicators of non-neural development are shown in Fig. 6E and 6F. Fig. 7 shows ECM component expression (Fig. 7A and 7B), ECM receptor expression (Fig. 7C and 7D), indicators of apoptosis (Fig. 7E and 7F) and Wnt canonical pathway (Fig. 7G and 7H), similarly compared to Day 0 in the left column and Day 8 in the right. Sex-determining region Y-box2 (Sox2) may be indicative of NSCs in the adult brain as well as regionally specific during embryonic development [43]. Sox2 was present at all times tested, but did not show a relatable pattern in its expression during culturing (data not shown). Pax6 and TBR2 were present in all samples tested from Day 4-Day 14 (data not shown). TBR1 was not detectable.

In addition to presence of NSCs, the increase in expression of PDGFRα and GFAP through Day 14 (Fig. 6A) likely represents an increase in the number of mature oligodendrocytes and astrocytes in our cultures, consistent with the overall increase in markers of mature neural phenotypes (Fig. 6B). An increase in many ECM components and their receptors following Day 8 is evident in Fig. 7A and 7B. An increase in Akt1 expression (Fig. 7E) and decrease in Trp53 expression (Fig. 7E) are consistent with a steady but increasing level of caspase-3-mediated apoptosis during niche maturation (Fig. 7E and 7F).

To compare the cells of the mature *in vitro *NSC niche to those immediately following retinoic acid induction, we analyzed gene expression on Day 10, 12 and 14. This comparison is shown in its entirety in Additional file 7. Genes that maintained a statistically significant (p < 0.05) difference in expression when compared to Day 8 are shown in the right panels of Fig. 6 and 7. Expression comparisons suggested continued neurogenesis between Day 8 and Day 14. GFAP expression increased from Day 10 through Day 14 (Fig. 6B), while mature neural markers increased greater than 10 fold during this same period (Fig. 6D). Decreased alpha fetoprotein expression following a Day 8 spike in expression (Fig. 6E) was consistent with a steady decrease predicted based on observations during murine development after initial hepatic lineages are determined [44]. The decrease in Pecam1 expression (Fig. 6E and 6F) correlated with the application of retinoic acid [45]. During niche maturation, syndecan -3, collagen IV and nidogen 1 were up-regulated (Fig. 7A and 7B) along with receptors VE-cadherin (cdh5), N-cadherin (cdh2) and β-1 integrin (Itgb1) (Fig. 7B and 7D), consistent with gene expression during the development of a NSC niche environment [17,45]. Nid2 expression is largely unchanged during niche formation, which is in agreement with the complementary nature of nidogen 1 & 2 in basement membrane formation [46-49]. Finally, increased expression of bone morphogenic protein 4 (Bmp4) (Fig. 7H) may be responsible for driving the GFAP^+ ^cell population increase [50],.

### Flow cytometric analysis supports evidence of NSCs in the niche

**Figure 8:**
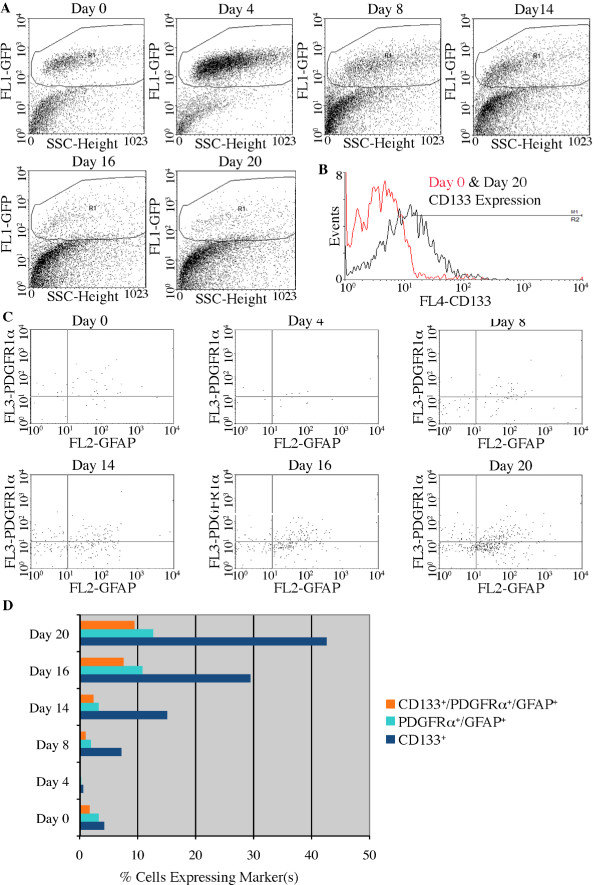
**Cell sorting analysis demonstrates the presence of putative NSCs resident in the *in vitro *NSC niche**. **A**, Gates for "live" cells included dot plot of side scatter (ssc) versus GFP fluorescence (FL1-GFP). The cellular population was fairly uniform in scatter on Days 0-8, but showed greater variety in Days 14-20 as the GFP^+ ^cells adopt their mature cell-fates. **B**, There is an increase in the number of CD133 labeled cells on Day 20 when compared to Day 0. **C**, Cells gated through the live gate in (**A**) and the CD133^+ ^gate in (**B**) are further analyzed for PDGFRα and GFAP expression. The upper right quadrant indicates cells that are positive for all three markers. **D**, Percentages of cells on different days of culture that express CD133, PDGFRα and GFAP, or all three markers. Day 0 represents undifferentiated mouse embryonic stem cells in culture; Days 4 and 8 are taken from cells during the 4-/4+ neural induction; Days 14, 16 and 20 are taken from cells in the *in vitro *NSC niche after the 4-/4+ neural induction.

We detailed previously the identification of NSCs within our culture sytem via co-expression of PDGFRα and GFAP (an indicator of NSCs in the adult SEZ [6]) within our culture system [6,9]. CD133 is a transmembrane protein often used to identify NSCs [38], but does not consistently identify the putative NSC in the SEZ after embryonic development [51]. To determine if the NSC in the *in vitro *NSC niche expresses CD133, we used flow cytometry to identify the stem cells during niche maturation from Days 0-14 and at Day 20 (Fig. 8). Although most cells that co-expressed PDGFRα and GFAP also expressed CD133 (Fig. 8D), many cells expressed CD133 with only one of the other markers or with neither. The latter group may represent embryonic ependymal cells [51] or even cells of a hematopoietic lineage [52], considering the high levels of VE-cadherin expression in the cultures (Fig. 7C and 7D). There was an increase in total number of NSCs in the cultures over time (Fig. 8D), but the ratio of NSCs to other cells in the culture is not likely accurately portrayed in flow analysis due to preferential loss of mature cell phenotypes (those with complex processes) during dissociation of the cultures for flow cytometry. The mature, morphologically complex cells may be lost during mechanical dissociation, while cells with simple cellular morphologies (including stem cells) are more likely to survive mechanical dissociation.

## Discussion

In this study, we describe the unique properties of a putative *in vitro *NSC niche. We previously defined a set of criteria that an *in vitro *system should satisfy to appropriately model elements of the developing and adult subventricular *in vivo *NSC niche [9]. These properties include the presence of NSCs; production of signaling molecules characteristic of NSC niches; the presence of transit-amplifying cells; the ability to produce both neurons and glia; the presence of a basal lamina and extracellular matrix; the contribution of radial glia as the primitive source of stem cells and as a scaffold for organization of the niche microenvironment; and autonomous production of cellular and molecular factors necessary for self-renewal and differentiation of resident stem cells. Because our mature culture system exhibits all of these characteristics, we propose that it is an *in vitro *NSC niche.

Our electron micrographs show the complexity of the aggregates and the strands of many cable-like processes between them (Fig. 1A-F). Based on our electron microscopy, we conclude that the *in vitro *NSC niche is a complex network of interconnected aggregates, each of which contains presumptive stem cells capable of differentiation. This observation supports our earlier findings that NSCs and their progeny are maintained within the *in vitro *NSC niche [9].

Although there is considerable cellular heterogeneity in the *in vitro *NSC niche, the cultures do retain a consistent molecular signature. First, the initial retinoic acid induction [34] causes the niche to become predominantly neural, consisting of a diversity of cells from NSCs to mature neurons, glia, and oligodendrocytes (Figs. 2, 6 and 8) [9]. However, there is continued evidence of non-ectodermal lineages present in the cultures (Fig. 6E). Though there is a steady increase in expression of genes indicative of mature neural cells between Days 10 and 14 (Fig. 6A and 6C), there remains a persistent presence of radial glia, NSCs and transit-amplifying cells in the cultures (Figs. 2, 6 and 8). A clear progression of expression of Pax6 through TBR2 to TBR1^+^neurons is indicative of neurogenesis in the SVZ [22,23]. The presence of Pax6 and TBR2 expression may suggest this progression in our cultures. However, there is not a clear and significant increase or decrease in these transcripts during culturing.

The development of the *in vivo *subventricular NSC niche is dependent on a network of ECM components [17,18,53] and their receptors [3,4,10,17]. Likewise, cells of the *in vitro *NSC niche express the specific ECM components suggestive of stem cell maintenance including the gamma-1 subunit of laminin, nidogen 1, collagen IV, perlecan, and syndecan-3 (Fig. 2C, Fig. 7A and 7C). Expression of E, P, VE, and N-cadherins suggests that there are cells within the *in vitro *NSC niche representing various levels of differentiation and lineage commitment, and may further suggest a mechanism for the organization of the aggregates based on cadherin expression. It is possible that the level of lineage commitment of neural progeny determines the probability of their association with different locations in the niche [20]. β-1 integrin acts to regulate stem cell identity and anchor the putative NSC to the ECM [10,17,52]. β-1 integrin expression in the *in vitro *NSC niche suggests that it may retain these roles in our cultures (Fig. 2C, Fig. 7C). Wnt1 and Bmp4 expression (Fig. 7G) are likely involved in additional stem cell regulation, having been implicated in self-renewal pathways [1,54].

The maintenance of a NSC population within the *in vitro *NSC niche is strongly supported by our protein expression data (Fig. 8). There is a NSC population that co-expresses PDGFRα, GFAP and CD133, possibly indicating a transition from embryonic NSC to adult NSC in neural cultures [6,51]. Cells that express CD133 but not PDGFRα or GFAP may be indicative of early embryonic NSCs, adult ependymal cells, or from a non-neural lineage. High expression levels of VE-cadherin on Day 14 may indicate that some CD133^+ ^cells are of a hematopoietic lineage [52]. Interaction with cells of a hematopoietic lineage within our cultures could be necessary for NSC and niche maintenance [12-14].

Development of the *in vitro *NSC niche appears to be more dependent on density (Additional file 2), than exogenous ECM application (Fig. 3 and Additional file 3). The cues for aggregation of the cells are not known, but β-1 integrin and other ECM components and receptors likely contribute to the aggregate formation, process extension, and cellular migration within the developing niche cultures [9,20,55].

Apoptosis occurs concurrently with the development and reorganization of the *in vitro *NSC niche, and a role for apoptosis has been demonstrated in this development *in vivo *[24,56]. Our data suggest that primarily the mature cells of the *in vitro *niche exhibit apoptotic activity (Fig. 5). This may be due to metabolic stress induced by serum deprivation, or may relate more directly to the presence or absence of downstream neural connectivity [24]. Varicosities on processes and cytoplasmic blebbing (Fig. 1E) also suggest a role of apoptosis in maturation of intercellular networks and remodeling of the niche cultures [24,57].

Here we have described the morphological properties of the *in vitro *NSC niche, the molecular components produced by cells of the niche cultures, the maintenance of a NSC population in the niche and the persistence of neurogenesis. We previously described use of the 4-/4+ retinoic acid neuralization protocol [34] in preparation of neuralized ES cells for transplant into mouse models of neurodegeneration [35,58]. Meyer et al. [35] described few Nestin-expressing neural precursors present when neuralized ES cells are dissociated and plated at low densities for 8 days as adherent cultures. Retinoic acid induction promotes neural differentiation of ES cells and additional time in culture favored differentiation of post-mitotic cells [9,34,35].

The protocol used to produce the *in vitro *NSC niche enriches for NSCs while retaining the potential for further neurogenesis (Fig. 8), and neurogenic potential appears to be increased significantly when compared to retinoic acid induction alone (Fig. 6B and 6D). Smith and colleagues have described a method of ES cell neuralization that results in a very homogeneous culture of NSCs [27]. With regard to transgenic modifications of a stem cell culture, homogenous populations may more easily be transduced with an expression vector of interest [59]. In cell culture, it is possible to direct neural fate specification through substrate selection [19], but it may be difficult to maintain broad potential for differentiation from a homogeneous pool of precursors [60].

There are complex molecular and cellular interactions that regulate NSCs in the *in vivo *NSC niche [1,3,4]. Initial efforts in isolation and passage of primary NSCs from developing mammalian brain were achieved using neurospheres, floating clusters including NSCs, neurons, and glia [61]. NSCs within neurospheres retain competence for self-renewal and are multipotent [62], but the cell-types present within neurospheres may change with repeated passage [63]. If neurospheres are allowed to adhere in close proximity they exhibit unique properties, such as migration of cells between clusters along complex processes. ffrench-Constant and colleagues suggested that a culture of adherent neurospheres with cells migrating between them was similar to SVZ explants [55]. Likewise, there are similarities in morphology, cell-types, and cellular migration in our ES-derived *in vitro *NSC niche, neurospheres in adherent cultures, and the SVZ NSC niche [9].

The *in vitro *NSC niche is the first neurogenic culture system to be produced without the application of exogenous mitogenic factors and complex physical scaffolding. Although the roles in niche formation of endogenous factors that influence the derivation and maintenance of this the *in vitro *NSC niche microenvironment have yet to be tested, our model is able to recapitulate many characteristics of the previously described *in vivo *NSC niche. Therefore, the *in vitro *NSC niche may be a valuable tool for assaying interactions within the *in vivo *SVZ or SEZ niche.

Exogenous application of ECM and the 3-dimensional nature of aggregates in the *in vitro *NSC niche provide depth to the culture system that appear to be required for growth factor production, cell migration [9,64] and stem cell maintenance (Fig. 8D). We addressed whether 3-D scaffolding might give additional benefit in bringing the *in vitro *NSC niche more fully into the third dimension [65-68]. To that end, we chose to use PuraMatrix hydrogel scaffolding system. First, PuraMatrix hydrogel is comprised entirely of polymeric amino acids and does not require serum or any components of serum. Our system forms in the absence of mitogens from Day 8-14. Any serum component added with a matrix could result in differentiation of the resident NSCs prior to niche formation. Second, the concentration of the RADA-16 component of PuraMatrix can be adjusted to be permissive to neurite outgrowth [69]. Third, PuraMatrix "self-assembles" and the use of a defined component, consisting of only amino acids, provides structure without unknown components. Finally, the ability to add binding sites specific to the promotion self-renewal or differentiation [70] offers the possibility of deriving a unique scaffold optimized for niche growth.

We have been able to reproduce niche morphology of the 2-D system in 3-D PuraMatrix when a 10-fold increase in ECL concentration is used (Fig. 3), but the pattern of mRNA expression by the niche changed in PuraMatrix (Fig. 4). Both 2- and 3-D cultures were capable of integration and survival in organotypic slice cultures, suggesting they are a possible source for cells in transplant studies. Further work is required to optimize the 3-D culture methods and characterize the cellular outcomes before a 3-D NSC niche can be widely used for transplantation.

The *in vitro *NSC niche may provide a novel source of cells for therapeutics. Because this culture system includes CD133^+^/PDGFRα^-^/GFAP^- ^NSCs with properties in common with embryonic NSCs [51] along with CD133^-^/PDGFRα^+^/GFAP^+ ^NSCs similar to those of the SEZ [6] (as well as a possible transitioning population of CD133^+^/PDGFRα^+^/GFAP^+ ^cells), it may have a broader potential for therapeutic value than any single cell population (Fig. 8D). It will likely provide a source of cells for transplant able to adhere to the ECM expressed by recipients of a wider range of ages. Furthermore, as the *in vitro *NSC niche is derived from an ES cell population, transgenic modification can be accomplished followed by clonal expansion [59].

## Conclusions

The *in vitro *NSC niche shows an identity unique from the 4-/4+ neurally-induced cells that are used as starting components for its derivation. Many molecular and cellular components consistent with a NSC niche were demonstrated in this system, including NSCs and their progeny, ECM components (both applied and endogenously produced) and their receptors, and the presence of apoptosis. Applications of this culture system include studies of the neurotoxin effects on brain development as well as NSC niche transplantation to treat disease or injury. Because the brain is not a homogenous cellular system, a complex, multicellular system that mimics the *in vivo *NSC niche may offer a superior model for studies of neural function and as a platform for cell-based therapies.

## Methods

### Cell Culture

B5 mouse embryonic stem cells (ESCs) (obtained from Dr. Andras Nagy, Samuel Lunenfeld Research Institute, Mt. Sinai Hospital, Toronto, Ontario, Canada) were passaged as previously described [9]. Briefly, ESCs were maintained on gelatin in a feeder-free environment with application of leukemia inhibitory factor (LIF) (Chemicon cat. # ESG1106) in cell culture medium containing 20% serum. Initial neural induction was performed as described previously [9,34,35,58] with the addition of all-trans retinoic acid (Sigma-Aldrich cat. # R2625) to embryoid bodies in suspension culture in the absence of LIF. Following retinoic acid induction (on Day 8 of culture), cells were plated at a cell density of 250,000 cells per cm^2^on the entactin-collagen-laminin (ECL) (Upstate cat. # 08-110) coated surface required of each experiment. Cells for the density experiment were plated at the densities reported, ranging from 1.25 × 10^5 ^cells/cm^2 ^to 2.0 × 10^6 ^cells/cm^2^. Culture medium for attachment cultures following 4-/4+ included Dulbecco's Modified Eagle Medium (DMEM; Hyclone cat. # SH30022.02), N2 supplement (Invitrogen cat. # 17502-048), penicillin-streptomycin (100 ug/ml) (Gibco cat. # 15140-122) and 250 μg/ml Amphotericin B (Gibco cat. # 15290-018). For immunohistochemistry and density experiments, cells were grown in 8-well culture slides (BD Falcon cat. # 354108) on 6.0 μg/cm^2 ^ECL. For RT-PCR and FACs experiments, cells were grown in T25 culture flasks (Corning cat. # 3055) on 6.0 μg/cm^2 ^ECL.

For 3-D versus 2-D comparison cultures, PuraMatrix (1% w/v; 3DM, Inc., Cambridge, MA, http://www.puramatrix.com/company.html) stock solution was diluted to 0.15% by the addition of sterile dH_2_O per protocol described in the PuraMatrix Guidelines. Diluted PuraMatrix was placed into the wells of a 96-well plate (Becton Dickinson cat. # 353072) at 50 uL/well. Culture medium was combined with ECL, laminin (BD cat. # CB40232), or collagen (BD cat. # CB 40233) at a ratio resulting in reported final concentrations for each well and slowly added to promote assembly of the PuraMatrix. The plate was incubated at 37°C for one hour to achieve equilibration. The media was then drawn off above the PuraMatrix and 100 uL of culture media was added to each well. The plate was incubated at 37°C for one hour and then changed again and left overnight at 37°C. Media was drawn off before using the plate. Day 8, embryoid bodies (EBs) were dissociated and resuspended in DMEM with N2 supplement at 1000 cells/uL. The cell suspension was carefully added to the top of the PuraMatrix and incubated at 37°C. On Days 9, 11, and 13, the media was drawn off above the PuraMatrix and replaced with 50 μL/well of fresh N2 media. 2-D comparative cultures were grown in the same 96-well plate, but wells were coated with appropriate concentrations ECM components without PuraMatrix.

Phase-contrast images for analysis of the effect of density on *in vitro *NSC niche formation were obtained using an Olympus IX70 microscope equipped with a Photometric Sensys CCD camera and Openlab v. 2.2.5 software. Light microscopy of 3-D comparison experiment was performed with transmitted light on the Zeiss LSM 510 M-200 AXIOVERT NLO 2-Photon confocal microscope and images processed using LSM 5 Image Examiner software and Adobe Photoshop CS.

### Electron Microscopy

On Day 8, neuralized ES cells were cultured on ECL coated thermanox coverslips (Thermonox, Nalgene Nunc Cat. # 174950) to facilitate optimal sectioning. The cells were placed in a humidified incubator containing 5% CO_2 _and media changed every other day until structure was apparent on Day 14 as described earlier. On Day 14, the cells were fixed in 2% glutaraldehyde (Polysciences, Inc. Cat. # 01909-10), 2% paraformaldehyde (EMD Chemicals Cat. # PX0055/3) in 0.1 M sodium cacodylate buffer at pH 7.4. All EM samples were processed according to a previously described microwave-assisted protocol [71] in a Pelco Biowave microwave under vacuum.

The cells were washed in 2-mercaptoethanol (ME) buffer (0.1 M sodium cacodylate, 0.13 M sucrose, 0.01 M 2-ME) and post-fixed in 1% osmium tetroxide (Electron Microscopy Sciences Cat. # 19160) in 2-ME buffer. The post-fixed cells were then dehydrated in a graded series of ethanol exchanges to a final dehydration in 100% ethanol. For TEM, the ethanol-dehydrated samples were rinsed with 100% acetone then infiltrated with Epon/Spurr's resin, equal parts Epon and Spurrs resins (Electron Microscopy Sciences Cat. #'s 14900 and14300). Samples were sectioned at 85 nm thickeness and stained with 5% uranyl acetate (Electron Microscopy Diatome Cat. # 22400) and Sato's triple lead stain [72] to enhance contrast before viewing on a JEOL 1400 Transmission Electron Microscope. Additional staining to enhance membrane contrast included adding ferrocyanide (EMD Chemicals Cat. # PX1460/1) during the post-fixation steps. For SEM, after ethanol dehydration, the samples were critical point dried and sputter-coated with platinum before viewing on an Hitachi S4700 Field Emission Scanning Electron Microscope.

### RT-PCR

Total RNA was purified from pelleted cells (for 3-D cultures, cells were prepared following PuraMatrix manufacturer recommendations for diluting matrix and pelleting cells) for reverse transcriptase PCR using Sigma's GenElute Mammalian Total RNA Miniprep Kit (Sigma-Aldrich cat. # RTN-70) as instructed by the manufacturer. A total of 45 μl isolated RNA was treated with five units of Promega's RQ1 RNase-free DNase I (Promega cat. # M610A) and 5.5 μl of 10× Reaction Buffer (Promega cat. # M198A) and incubated at 37°C for 30 min. DNase I was inactivated with 6 μl of Stop Solution (Promega Cat. # M199A) and incubated at 65°C for 15 min. The cDNA and corresponding No Reverse Transcriptase (No RT) negative controls were synthesized using Marligen's First-strand cDNA Synthesis kit (Marligen cat. # 11801-100) according to the protocol provided by the manufacturer. PCR was performed using Eppendorf's 2.5× Hot-MasterMix (Eppendorf Cat. # 954140181) and custom oligonucleotides from either Integrated DNA Technologies (IDT, Coralville, IA) or Sigma's Genosys. Controls lacking reverse transcriptase were pooled prior to PCR and proved to be negative (data not shown).

Oligonucleotides were designed with the SciTools software provided by Integrated DNA Technologies using the indicated FASTA sequence from Entrez Nucleotide. Reference Sequences and product sizes are reported in Additional file 1. The PCR parameters used were 94°C for 30 sec followed by 35 cycles of 94°C for 20 sec, 50°C for 30 sec, and 70°C for 45 sec with a final elongation time of 5 min at 70°C. PCR products were run on agarose gels by standard electrophoresis techniques and detected using a Kodak Gel Logic 200 imaging system along with Kodak Molecular Imaging software.

### Immunohistochemistry

Culture slides were collected on Day 14 of induction, and the cells were fixed for 20 minutes in 4% paraformaldehyde in phosphate-buffered saline (PBS). The cells were washed thoroughly (3 × 5 min) with 0.1 M PBS and then permeabilized for one hour at room temperature with 1% Triton X-100 in 0.1 M PBS. The cells were then washed thoroughly (3 × 5 min) with blocking solution (0.1 M PBS, 2% normal goat serum (Invitrogen Cat. # 16210-064), pH 7.4) at room temperature. TUNEL labeling was performed using the *In Situ *Cell Death Detection Kit, TBR red (Roche cat. # 12 156 792 910) as directed by the manufacturer. Primary antibodies selective for early neurons (mouse monoclonal for β-III Tubulin, 1:200, Chemicon cat. # MAB5544), late neurons (rabbit polyclonal for Neurofilament-M, 1:200, Chemicon cat. #AB1981) and apoptosis (rabbit polyclonal for cleaved caspase-3 (Asp175), 1:250, Cell Signaling Technology cat. # 9661) were diluted in blocking solution and applied overnight at 4°C. Appropriate fluorescent tagged goat anti-rabbit (F(ab')2 fragmented, Molecular Probes cat. # A21072) and goat anti-mouse (Molecular Probes cat. # A11004) secondary antibodies were diluted in blocking solution and applied individually for 2-4 hours at room temperature. Nuclei were stained with DAPI (1:300, Molecular Probes, cat. # D3571). Cells were imaged using a Zeiss LSM 510 M-200 AXIOVERT NLO-2-Photon confocal microscope (Molecular Cytology Core, University of Missouri). Images were processed using LSM 5 Image Examiner software version 4.0.0.157 and Adobe Photoshop CS.

### Quantitative RT-PCR

Total RNA was purified from pelleted cells for quantitative real time PCR using Qiagen's RNeasy Mini Kit (Qiagen cat. # 74104) according to the manufacturer's instructions. Cell lysates were homogenized as directed with Qiagen's QIAshredder spin columns (Qiagen cat. # 79654) that also sheared larger genomic DNA. The optional DNA digestion was performed on the RNeasy columns during RNA purification using Qiagen's RNase-free DNase Set (Qiagen Cat. # 79254) as suggested by the RNeasy Mini Kit instructions. One exception was made to the protocol. The DNA digestion was allowed to continue at room temperature for 30 minutes, as opposed to the recommended 15 minutes, as proposed by SuperArray's Technical Service. RNA quantification was performed on a NanoDrop ND-1000 UV-Vis Spectrophotometer, which also confirmed the quality of each sample.

SuperArray's RT^2 ^First Strand Kit (SuperArray Cat. # C-03), which includes a genomic DNA elimination step, was used to synthesize cDNA from each RNA sample as directed by the manufacturer. Pooled negative controls were made in similar reactions without reverse transcriptase. qRT-PCR was performed, using the resultant cDNA and No RT negative controls and SuperArray's RT^2 ^SYBR Green/Flourescein qPCR Master Mix (SuperArray Cat. # PA-011-12) in custom primer plates from SuperArray according to SuperArray's instructions, on a BioRad iQ Real-Time PCR Detection System.

To determine the optimal, positive control, housekeeping gene for qRT-PCR experiments, cDNA samples that were also used as experimental cDNA were amplified as suggested by the manufacturer with SuperArray's Mouse Housekeeping Genes RT^2^*Profiler*™ PCR Array (SuperArray Cat. # PAMM-000) that contains twelve of the most commonly used mouse housekeeping genes. The range and standard deviation were calculated from the threshold (Ct) values generated by each prospective housekeeping gene (Additional file 6). The TATA box binding (TBP) housekeeping gene had the lowest range and standard deviation, each about half of the next nearest housekeeping gene. Based on this data, TBP was chosen as the positive control, housekeeping gene for all qRT-PCR assays. Experimental reactions were carried out as directed by SuperArray, and post-run melt curve analysis for each negative control reaction confirmed the absence of contaminating DNA (data not shown). Each experimental and positive control qRT-PCR reaction was run in triplicate (n = 3) with RNA for each of the three replicates coming from separate cultures of cells. The fold changes were calculated using ΔΔCt method. To determine statistical significance of fold-change data, a student T-test was performed, assuming a two-tailed sample distribution and equal variance.

### Flow Cytometry

Attached cells from Days 0, 14, 16 and 20 were fully dissociated with TrypLe (Invitrogen Cat. # 12604-013) (5 min at 37°C) and cells from Days 4 and 8 embryoid bodies were pelleted, washed with PBS and dissociated with TrypLe (8 min at 37°C). The enzyme was quenched with PBS containing 5% FBS (Atlanta Biologicals cat. # S1150). Cells were passed through cell strainer tubes (BD Falcon Cat. # 352235) and brought to a concentration of 4,000-5,000 cells/μl. Labeling, permeabilization and fixation was performed as recommended by Santa Cruz Biotechnology, Inc., using FCM wash buffer (Santa Cruz Cat. # sc-3624), FCM permeabilization buffer (Santa Cruz Cat. # sc-3623), and FCM fixation buffer (Santa Cruz Cat. # sc-3622). Prior to permeabilization, 1 μg of Allophycocyanin conjugated primary antibody rat-anti-mouse prominin-1 (Miltenyi Biotec Cat. # 130-092-335) and biotinylated primary anti-mouse CD140a (PDGFRα)

### Animal Care and Use

All procedures using animals for research were approved by the Office of Animal Resources, 1720 East Campus Loop, University of Missouri, Columbia, MO 65211. The protocol number for this work is 4219.

## Authors' contributions

CP conceived of the study, participated in its design, performed cell culture, performed light, phase, and confocal microscopy, performed flow cytometry, constructed all figures and tables, and helped to write the manuscript. JAM (Jason Morrison) participated in the design of the study, performed all RT-PCR and quantitative RT-PCR, performed cell culture, performed light and confocal microscopy, and helped to write the manuscript. PR performed electron microscopy. REZ performed 2-D and 3-D cell culture. LAE performed immunohistochemistry. CLF performed TUNEL labeling. HS participated in design of the study and analyzed quantitative RT-PCR data. JAM (Joel Maruniak) participated in the design of the study and helped to write the manuscript. MDK participated in the design of the study, managed the project, and helped to write the manuscript.

## Supplementary Material

Additional file 1 **RT-PCR genes and primers**. Genes, Reference Sequences (RefSeq), Primers, and Amplicon information are given for the RT-PCR reactions. Click here for file

Additional file 2 **Phase microscopy of high density plating of cells following 4-/4+ retinoic acid neural induction**. A, Day 10 culture plated at 2 million cells/cm^2^. B, Day 10 culture plated at 1 million cells/cm^2^. C, Day 10 culture plated at 500,000 cells/cm^2^. D, Day 10 culture plated at 250,000 cells/cm^2^. E, Day 10 culture plated at 125,000 cells/cm^2^. F, Day 14 mature *in vitro *NSC niche plated at 250,000 cells/cm^2^. Though the Day 10 culture in (B) strongly resembles that of the Day 14 culture in (F), cells in the conditions shown in (A-C) failed to produce the equivalent of the *in vitro *NSC niche because at these high cell densities, the cells lifted off of the culture plate by Day 11. Cells shown in (E) failed to produce *in vitro *NSC niche because low-density plating prevented appropriate aggregate formation and favored cellular differentiation. Cells plated at 250,000 cells/cm^2 ^formed the mature niche depicted in (F). Scale bar in (A) is 50 μm in (A-E) and 100 μm in (F). Click here for file

Additional file 3 **Light microscopy of 2- and 3-D cultures grown in the presence of various ECM components**. **Ai-Gi**, Following 4-/4+ retinoic acid neural induction of mouse embryonic stem cells, the cells are plated in 96-well plates at 250,000 cells/cm^2 ^on the indicated substrate. Images are from Day 10 cultures. **Aii-Gii**, Cells are plated in 3-D using 0.15% Puramatrix hydrogel with the addition of the ECM substrate indicated. Images are from Day 10 cultures. **Hi-Oi **and **Hii-Oii**, 2-D and 3-D cultures, respectively, on Day 12 of culture development. Scale bar in **(Ai) **is 100 μm and applies to all panels. *Concentrations of ECM components are expressed per surface area of the culture well. ECM components for 3-D cultures are added at the same concentration as 2-D cultures. The volume of PuraMatrix assembled from 50 μl of 0.15% PuraMatrix in each well increases the surface area for ECM attachment, thus diluting the effective concentration of the ECM components. In all panels, arrows indicate process development and arrowheads indicate compact aggregate formation. Click here for file

Additional file 4 **Transplantation of *in vitro *NSC niche in 2- and 3-D onto organotypic slice cultures**. **A-C**. Integration of *in vitro *NSC niche cultures into organotypic brain slice cultures. Organotypic cultures were prepared and imaged as previously described [73-75]. **A**, Phase contrast image of a 400 μm slice culture taken four days after harvest from a postnatal day 8 mouse pup. **B**, On Day 14, 2-D *in vitro *NSC niche cultures were dissociated in 5% Trypsin and applied to the organotypic slice cultures. Serum-free media was changed once a week. Transplanted cells were imaged 8 days after transplantation with a Leica MZFLIOII stereomicroscope equipped with epi-fluorescence. **C**, Intact 3-D cultures at Day 14 were drawn from the well with a 1000 μl pipet tip and applied to the organotypic slice culture as in (**B**). Click here for file

Additional file 5 **Gene information**. Reference Sequences (RefSeq), gene symbols and names are given for quantitative RT-PCR reactions. Click here for file

Additional file 6 **Housekeeping gene selection**. Quantitative RT-PCR data for SuperArray housekeeping gene selection are given. Expression of potential housekeeping genes was determined at timepoints throughout niche formation and in the presence of serum and/or antioxidants. Serum and antioxidant addition was performed to further test the responsiveness and range of each housekeeping gene during culturing. For experiments including serum and antioxidant addition to culture medium, all methods are equivalent to previous cultures in T25 flasks (RT-PCR and FACs) until Day 11, when culture media includes either 20% serum (induction medium as previously described [35] and N2 supplement (Serum Culture Media), or additionally 1 mM N-acetyl Cysteine (NAC) (Fisher Scientific Cat. # 01049-25) (Serum/Antioxidant Culture Media). Standard deviation and range of threshold values (Ct) over the 14-day time-course were determined for 12 possible housekeeping genes. Because TATA Box binding protein (TBP) showed the lowest standard deviation and the smallest range, it was determined to be the most stabile housekeeping gene for our experiments. Click here for file

Additional file 7 **Fold change versus Day 8 control**. All Quantitative RT-PCR data are given for Days 10, 12, and 14 as fold-change from Day 8 controls. = indicates the value is significantly different from Day 8 value (p < 0.05). Click here for file
