# Supplementary material for: Developmental cues and persistent neurogenic potential within an in vitro neural niche
Source: BMC Dev Biol. 2010 Jan 14;10:5. doi: 10.1186/1471-213X-10-5 (PMC2824744; doi:10.1186/1471-213X-10-5)
Supplement: Additional file 1 — RT-PCR genes and primers. Genes, Reference Sequences (RefSeq), Primers, and Amplicon information are given for the RT-PCR reactions. [file 1471-213X-10-5-S1.PDF]

Gene	RefSeq	Forward Primer	Reverse Primer	Amplicon Length
PDFGRα	NM_011058	5 -ACATGTCAATGCCACGTATGCAGC- 3	5 -ATTCCCGGACACACTTTGAGGTGA- 3	194bp
GFAP	NM_010277	5 -TGCCACGTTTCTCCTTGTCTCGAA- 3	5 -TCGATGTAGCTAGCAAAGCGGTCA- 3	154bp
Ki-67	X82786	5 -AGGAGGAACCAACCAAGGACAGTT- 3	5 -AGTGTGTTCCTGGTTTCCCTGGAT- 3	230bp
BLBP	S69799	5 -TGAGTACATGAAAGCTCTGGGCGT- 3	5 -TGAGCTTGTCTCCATCCAACCGAA- 3	224bp
Map2a	M21041	5 -ATCCTGGTGCCCAGTGAGAAGAAA- 3	5 -GCCACATTTGGATGTCACATGGCT- 3	222bp
DAT	AF109391	5 -TTTGGAGGTTTCCCTACCTGTGCT- 3	5 -ATGAGGATGACAGTGAAGCCCACA- 3	196bp
TH	NM_009377	5 -TCACTGTGGAGTTTGGGCTGTGTA- 3	5 -TGTCCTTGGCATCACTGAAGCTCT- 3	213bp
NeuroD1	NM_010894	5 -AATTAAGGCGCATGAAGGCCAACG- 3	5 -TTTGCAGAGCGTCTGTACGAAGGA- 3	224bp
E-Cad/Cdh1	NM_009864	5 -AACAACTGCATGAAGGCGGGAATC- 3	5 -CCTGTGCAGCTGGCTCAAATCAAA- 3	246bp
P-Cad/Cdh3	NM_001037809	5 -ACACGACCTCATGTTCACCATCCA- 3	5 -ATCAAGGATTTGCACAACGGCCTC- 3	166bp
N-Cad/Cdh2	NM_007664	5 -AGAGCTGATAGCCCGGTTTCACTT- 3	5 -GGGCATTTGGATCATCCGCATCAA- 3	218bp
VE-Cad/Cdh5	NM_009868	5 -TCAGTTCCCTGAGCACCAATTCCT- 3	5 -AAATCAGGGAAGACAGTGTCCGGT- 3	204bp
Integrin α5	NM_010577	5 - TGTCTCCAAGCCTGAAGCTGTGAT- 3	5 -TAACTGGAGGTGCAGTTGCTGAGT- 3	234bp
Integrin β1	NM_010578	5 -CCCAAGTTTCAAGGGCCAACTTGT- 3	5 -TCTCCTTGCAATGGGTCACAGGAT- 3	214bp
Laminin γ1	NM_010683	5 -TCACCATGAAGGATCCCTTTCGCT- 3	5 -TTCACTCGATGCTCAGCAGCCTTA- 3	189bp
Nidogen 1	NM_010917	5 -ATGGCAAGGTGAAGGGAAGGATCT- 3	5 -TTCTTGAACCCATCCTGCTCCACT- 3	217bp
Nidogen 2	NM_008695	5 -ATCTACACGCTTACATCGTGGGCA- 3	5 -TCCACGTCATGGACAAAGGTAGCA- 3	193bp
Col IVα2	NM_009932	5 -GCCAAACGCACTTCCTGGAATCAA- 3	5 -CGGTGTTGCCCATGAATCCTTGTT- 3	170bp
Perlecan	M77174	5 -TATGTGTGCCGAGCCAACAACAAC- 3	5 -TGAGCCATGCGTCTGATGATGAGT-3	249bp
Caspase 3	NM_009810	5 -TGATGAGGAGATGGCTTGCCAGAA- 3	5 -AGAAGGACTCGAATTCCGTTGCCA- 3	218bp
RARα	NM_009024	5 -AGCTTCCAGTCAGTGGTTACAGCA- 3	5 -TGTTCTTCTGGATGCTTCGTCGGA- 3	219bp
RARβ	NM_011243	5 –TCATGAATAACCAGGCCTCACGGT- 3	5 -TCTGCTAGTGCAGTGAAGTGGTGT- 3	177bp
RARγ	BC013709	5 -AGGAACTCATCACCAAGGTCAGCA- 3	5 -CCGCTTCGCAAACTCCACAATCTT- 3	182bp
Prominin 1	NM_008935	5 -TGAATGACCACCTTGGAGACCGTT- 3	5 -AGTAACAGCAGGGCACTGAAGACA- 3	171bp
Dlx2	NM_010054	5 -TCCGAATAGTGAACGGGAAGCCAA- 3	5 -TCTTGAACTTGGATCGGCGGTTCT- 3	201bp
β-III Tubulin	NM_023279	5 -ATCCACCTTCATTGGCAACAGCAC- 3	5 -ACTCGGACACCAGGTCATTCATGT- 3	173bp
GAPDH	M32599	5 -TGTGATGGGTGTGAACCACGAGAA- 3	5 -AGTGATGGCATGGACTGTGGTCAT- 3	153bp
