# Supplementary figures and images for: Developmental cues and persistent neurogenic potential within an in vitro neural niche
Source: BMC Dev Biol. 2010 Jan 14;10:5. doi: 10.1186/1471-213X-10-5 (PMC2824744; doi:10.1186/1471-213X-10-5)

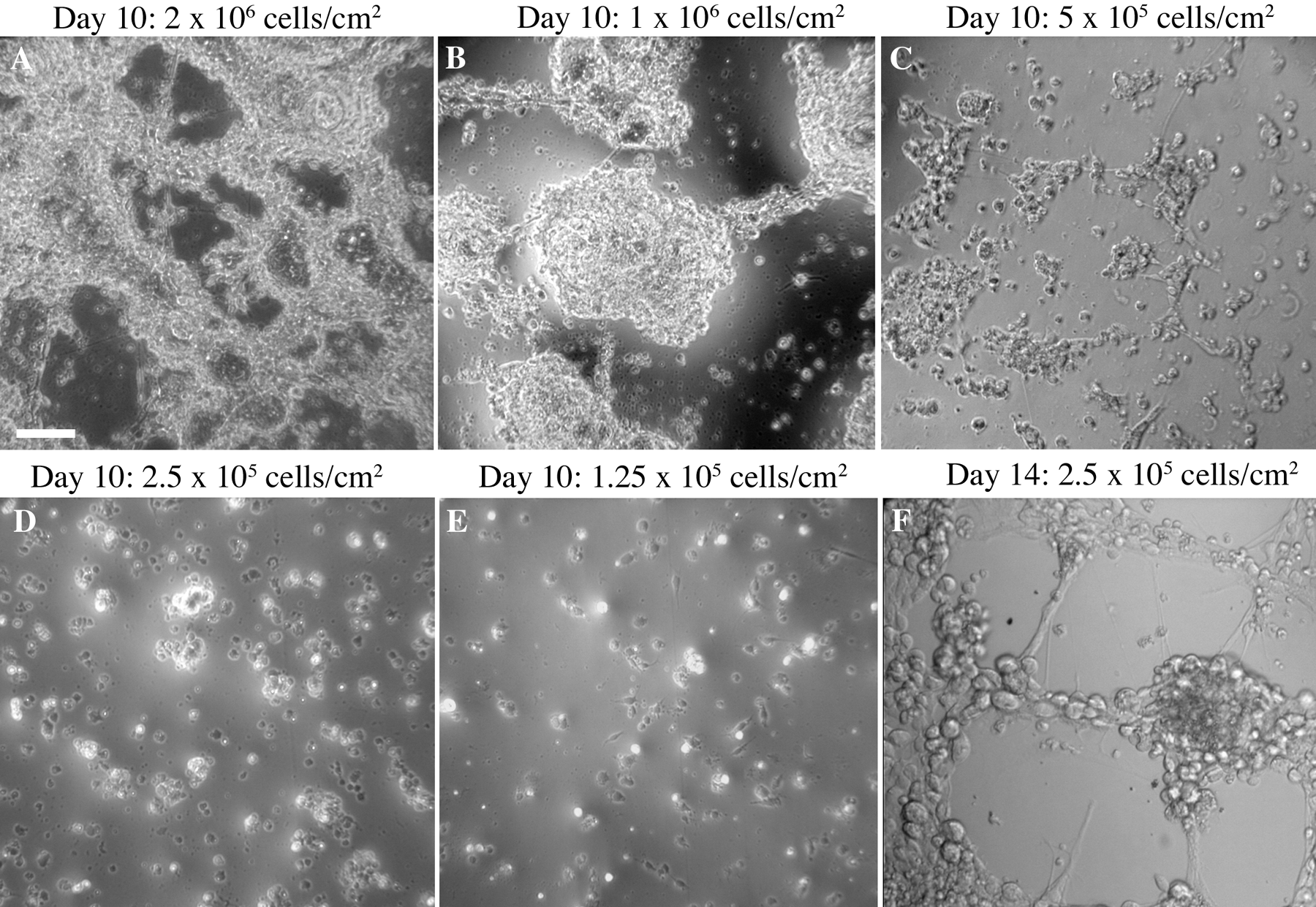

Supplement: Additional file 2 — Phase microscopy of high density plating of cells following 4-/4+ retinoic acid neural induction. A, Day 10 culture plated at 2 million cells/cm2. B, Day 10 culture plated at 1 million cells/cm2. C, Day 10 culture plated at 500,000 cells/cm2. D, Day 10 culture plated at 250,000 cells/cm2. E, Day 10 culture plated at 125,000 cells/cm2. F, Day 14 mature in vitro NSC niche plated at 250,000 cells/cm2. Though the Day 10 culture in (B) strongly resembles that of the Day 14 culture in (F), cells in the conditions shown in (A-C) failed to produce the equivalent of the in vitro NSC niche because at these high cell densities, the cells lifted off of the culture plate by Day 11. Cells shown in (E) failed to produce in vitro NSC niche because low-density plating prevented appropriate aggregate formation and favored cellular differentiation. Cells plated at 250,000 cells/cm2 formed the mature niche depicted in (F). Scale bar in (A) is 50 μm in (A-E) and 100 μm in (F). [file 1471-213X-10-5-S2.TIFF]

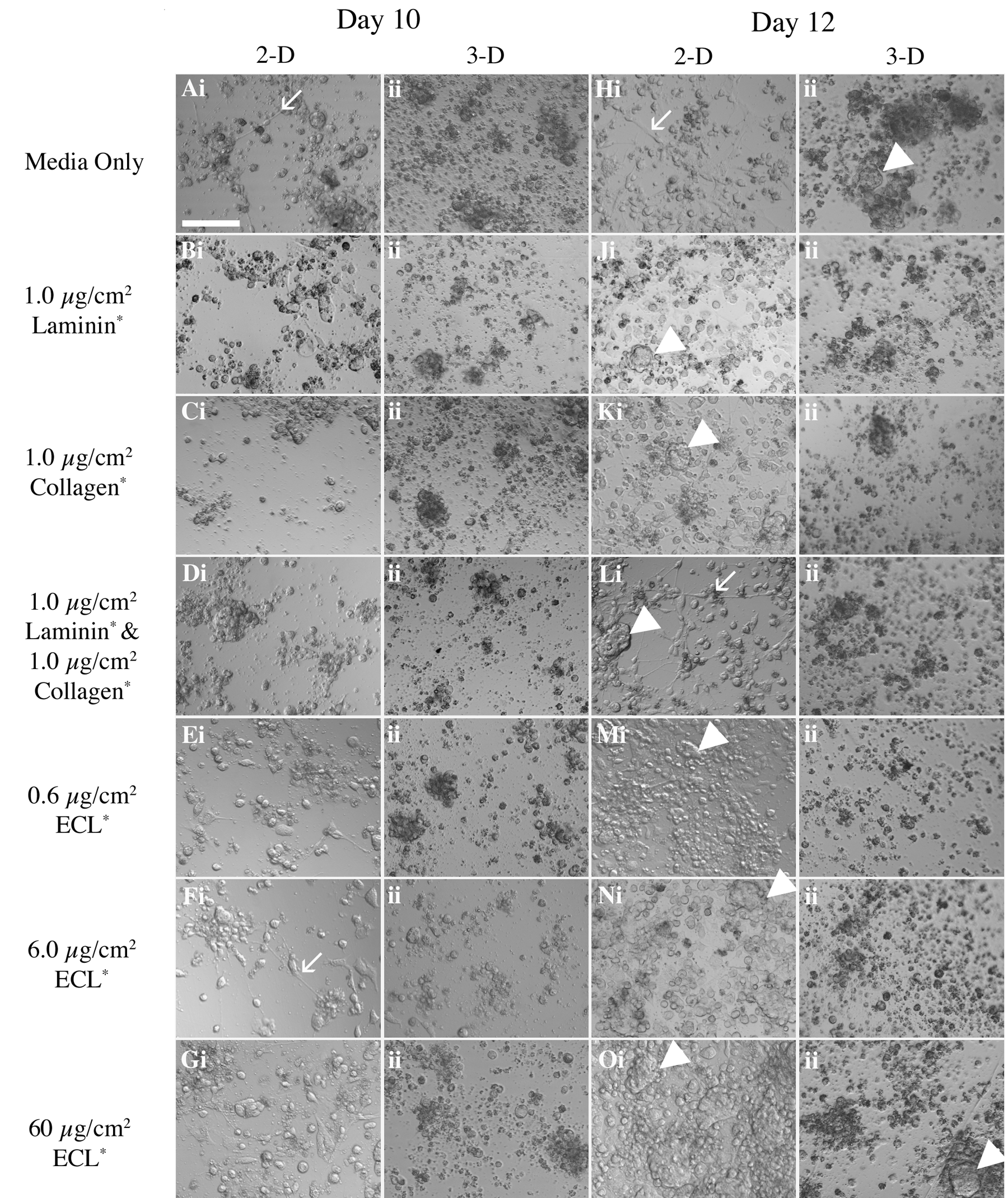

Supplement: Additional file 3 — Light microscopy of 2- and 3-D cultures grown in the presence of various ECM components. Ai-Gi, Following 4-/4+ retinoic acid neural induction of mouse embryonic stem cells, the cells are plated in 96-well plates at 250,000 cells/cm2 on the indicated substrate. Images are from Day 10 cultures. Aii-Gii, Cells are plated in 3-D using 0.15% Puramatrix hydrogel with the addition of the ECM substrate indicated. Images are from Day 10 cultures. Hi-Oi and Hii-Oii, 2-D and 3-D cultures, respectively, on Day 12 of culture development. Scale bar in (Ai) is 100 μm and applies to all panels. *Concentrations of ECM components are expressed per surface area of the culture well. ECM components for 3-D cultures are added at the same concentration as 2-D cultures. The volume of PuraMatrix assembled from 50 μl of 0.15% PuraMatrix in each well increases the surface area for ECM attachment, thus diluting the effective concentration of the ECM components. In all panels, arrows indicate process development and arrowheads indicate compact aggregate formation. [file 1471-213X-10-5-S3.TIFF]

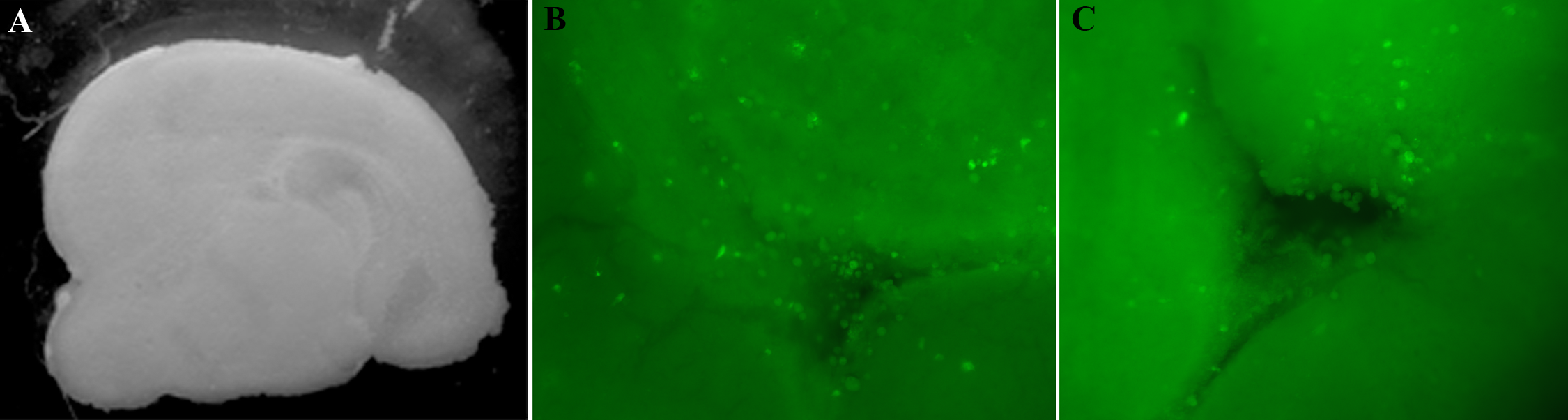

Supplement: Additional file 4 — Transplantation of in vitro NSC niche in 2- and 3-D onto organotypic slice cultures. A-C. Integration of in vitro NSC niche cultures into organotypic brain slice cultures. Organotypic cultures were prepared and imaged as previously described [73-75]. A, Phase contrast image of a 400 μm slice culture taken four days after harvest from a postnatal day 8 mouse pup. B, On Day 14, 2-D in vitro NSC niche cultures were dissociated in 5% Trypsin and applied to the organotypic slice cultures. Serum-free media was changed once a week. Transplanted cells were imaged 8 days after transplantation with a Leica MZFLIOII stereomicroscope equipped with epi-fluorescence. C, Intact 3-D cultures at Day 14 were drawn from the well with a 1000 μl pipet tip and applied to the organotypic slice culture as in (B). [file 1471-213X-10-5-S4.TIFF]
