# Supplementary material for: Developmental cues and persistent neurogenic potential within an in vitro neural niche
Source: BMC Dev Biol. 2010 Jan 14;10:5. doi: 10.1186/1471-213X-10-5 (PMC2824744; doi:10.1186/1471-213X-10-5)
Supplement: Additional file 5 — Gene information. Reference Sequences (RefSeq), gene symbols and names are given for quantitative RT-PCR reactions. [file 1471-213X-10-5-S5.PDF]

UniGene	RefSeq	Symbol	Description
Mm.1249	NM_010683	Lamc1	Laminin, gamma 1
Mm.4691	NM_010917	Nid1	Nidogen 1
Mm.20348	NM_008695	Nid2	Nidogen 2
Mm.738	NM_009931	Col4a1	Procollagen, type IV, alpha 1
Mm.273662	XM_978879	Hspg2	Perlecan (heparan sulfate proteoglycan 2)
Mm.206536	NM_011520	Sdc3	Syndecan 3
Mm.263396	NM_010578	Itgb1	Integrin beta 1 (fibronectin receptor beta)
Mm.34405	NM_009810	Casp3	Caspase 3
Mm.222	NM_011640	Trp53	Transformation related protein 53
Mm.6645	NM_009652	Akt1	Thymoma viral proto-oncogene 1
Mm.41329	NM_139001	Cspg4	Chondroitin sulfate proteoglycan 4
Mm.35605	NM_009864	Cdh1	Cadherin 1
Mm.4658	NM_001037809	Cdh3	Cadherin 3
Mm.257437	NM_007664	Cdh2	Cadherin 2
Mm.21767	NM_009868	Cdh5	Cadherin 5
Mm.1123	NM_021279	Wnt1	Wingless-related MMTV integration site 1
Mm.6813	NM_007554	Bmp4	Bone morphogenetic protein 4
Mm.291928	NM_007614	Ctnnb1	Catenin (cadherin associated protein), beta 1
Mm.913	NM_009309	T	Brachyury
Mm.343951	NM_008816	Pecam1	Platelet/endothelial cell adhesion molecule 1
Mm.247669	NM_008092	Gata4	GATA binding protein 4
Mm.358570	NM_007423	Afp	Alpha fetoprotein
Mm.3608	NM_013627	Pax6	Paired box gene 6
Mm.200692	NM_010136	Eomes	Eomesodermin homolog (Xenopus laevis)
Mm.308525	NM_009322	Tbr1	T-box brain gene 1
Mm.4541	NM_011443	Sox2	SRY-box containing gene 2
Mm.221403	NM_011058	Pdgfra	Platelet derived growth factor receptor, alpha
Mm.4636	NM_010894	Neurod1	Neurogenic differentiation 1
Mm.256966	NM_008632	Mtap2	Microtubule-associated protein 2
Mm.40068	NM_023279	Tubb3	Tubulin, beta 3
Mm.1239	NM_010277	Gfap	Glial fibrillary acidic protein
Mm.3896	NM_010054	Dlx2	Distal-less homeobox 2
Mm.6250	NM_008935	Prom1	Prominin 1, (CD133)
Mm.244820	NM_013684	Tbp	TATA box binding protein
