# Supplementary material for: Developmental cues and persistent neurogenic potential within an in vitro neural niche
Source: BMC Dev Biol. 2010 Jan 14;10:5. doi: 10.1186/1471-213X-10-5 (PMC2824744; doi:10.1186/1471-213X-10-5)
Supplement: Additional file 6 — Housekeeping gene selection. Quantitative RT-PCR data for SuperArray housekeeping gene selection are given. Expression of potential housekeeping genes was determined at timepoints throughout niche formation and in the presence of serum and/or antioxidants. Serum and antioxidant addition was performed to further test the responsiveness and range of each housekeeping gene during culturing. For experiments including serum and antioxidant addition to culture medium, all methods are equivalent to previous cultures in T25 flasks (RT-PCR and FACs) until Day 11, when culture media includes either 20% serum (induction medium as previously described [35] and N2 supplement (Serum Culture Media), or additionally 1 mM N-acetyl Cysteine (NAC) (Fisher Scientific Cat. # 01049-25) (Serum/Antioxidant Culture Media). Standard deviation and range of threshold values (Ct) over the 14-day time-course were determined for 12 possible housekeeping genes. Because TATA Box binding protein (TBP) showed the lowest standard deviation and the smallest range, it was determined to be the most stabile housekeeping gene for our experiments. [file 1471-213X-10-5-S6.PDF]

GENE :	18SrRNA	Actin, β	Microglobulin, β	Glucuronidase, β	Peptidylprolyl	Rpl13a
Sample:	Ct Value	Ct Value	Ct Value	Ct Value	Ct Value	Ct Value
Day 0	16	21.8	29	27.8	21	20.7
Day 0	16.5	22.2	28.8	27.7	20.6	20.8
Day 4	16.2	21.1	27.9	27	19.7	19.5
Day 4	16.2	21	27.6	26.9	19.8	19.6
Day 8	16.5	20.9	26.1	27.4	20.6	20.8
Day 8	16.8	21.2	26.6	27	20.6	20.7
Day 10	17.8	20.4	24.2	28.5	21.9	21.6
Day 10	17.5	20	23.8	28	21.1	21.5
Day 12	16.3	19.9	25.5	27.4	21.4	21.2
Day 12	16.6	20	25.7	27.8	20.7	21.2
Day 12 S	15.6	18.8	24.4	27.1	20.5	20.6
Day 12 SA	15.9	19.3	24.6	27.4	20.7	20.2
Day 14	17.1	19.8	24.6	27.7	20.9	21.1
Day 14	17.1	20	24.8	27.5	20.8	21.5
Day 14 S	16.8	19.4	23.8	26.8	20.4	20.5
Day 14 SA	16.1	19.4	23.9	27.2	20.6	20.8
Ct St Dev:	0.60	0.96	1.78	0.45	0.53	0.61
Ct Range:	2.2	3.4	5.2	1.7	2.2	2.1

GENE:	Hprt1	Hsp90ab1	Ldhal6b	Nono	TATA Box binding	Transferrin, r
Sample:	Ct Value	Ct Value	Ct Value	Ct Value	Ct Value	Ct Value
Day 0	33.2	21.7	32.3	27.1	28.1	25.6
Day 0	32.5	21.7	31.7	27.2	28.3	25.8
Day 4	31.4	20.1	32.8	26	27.8	24.9
Day 4	31.7	19.9	31.8	25.4	27.6	24.6
Day 8	31.8	21.8	32.5	26.6	27.9	26.3
Day 8	32.1	21.7	32.1	26.6	27.9	26.3
Day 10	33.1	23	35.2	27.7	28.4	27.6
Day 10	32.5	22.6	34.8	27.2	27.9	27.9
Day 12	32.4	22.2	34.5	26.6	28.3	26.8
Day 12	32.6	22.3	32.7	26.6	28.2	26.9
Day 12 S	31.4	21.1	33	25.8	27.7	25.6
Day 12 SA	32.3	21.5	32.2	26.3	27.6	25.5
Day 14	32.1	22.4	33	27	27.8	27.4
Day 14	32.1	21.9	32.4	27.4	28.1	27.3
Day 14 S	31.3	21.3	32.8	26.8	27.7	25.8
Day 14 SA	31.9	22	32.5	27.2	27.7	26.9
Ct St Dev:	0.56	0.82	1.04	0.62	0.26	0.98
Ct Range:	1.9	3.1	3.5	2.3	0.8	3.3
