# Supplementary material for: Developmental cues and persistent neurogenic potential within an in vitro neural niche
Source: BMC Dev Biol. 2010 Jan 14;10:5. doi: 10.1186/1471-213X-10-5 (PMC2824744; doi:10.1186/1471-213X-10-5)
Supplement: Additional file 7 — Fold change versus Day 8 control. All Quantitative RT-PCR data are given for Days 10, 12, and 14 as fold-change from Day 8 controls. = indicates the value is significantly different from Day 8 value (p < 0.05). [file 1471-213X-10-5-S7.PDF]

Symbol	D10	D12	D14
**Nid1**	4.59†	6.2†	4.29†
**Nid2**	0.87	0.60	0.42†
**Col4a1**	2.35	2.46	2.09†
**Hspg2**	2.24	1.95†	1.48
**Sdc3**	2.24†	3.73†	13.3†
**Itgb1**	1.10	1.95†	1.78†
**Cdh1**	2.46	1.05	0.87
**Cdh2**	1.10	3.25	7.82†
**Cdh5**	8.38	9.19†	14.25†
**Wnt1**	0.66	0.42	0.23
**Bmp4**	6.81†	7.29†	5.28†
**Ctnnb1**	0.74	0.68	0.68
**Pax6**	0.19†	0.30†	0.64
**Pdgfra**	2.64†	2.89†	3.56†
**Gfap**	0.72	2.05	32†
**Dlx2**	0.72	1.02	1.82
**Prom1**	0.66	0.74	1.59
**Cspg4**	4.09	6.5†	4†
**Mtap2**	1.18	8.57†	38.5†
**Tubb3**	2.70†	6.35†	17.14†
**Casp3**	0.51	1.29	2.46
**Trp53**	0.51†	0.55†	0.39†
**Akt1**	1.78	2.05†	1.78
**Pecam1**	0.22†	0.17†	0.06†
**Gata4**	4.29	3.10	2.64
**Afp**	0.13	0.02†	0.01†

† Value is significantly unique from Day 8 value ($P < 0.05$)

Genes included in table represent significant changes as noted. Genes tested which were not significantly unique from Day 8 include: Lamc1, Cdh3, T, Eomes, Tbr1, Sox2, Neurod1,
